# Antiretroviral Therapy Efficacy Post-Bariatric Weight Loss Surgery: A Case Series of Persons Living with Human Immunodeficiency Virus

**DOI:** 10.1007/s11695-022-05956-7

**Published:** 2022-02-16

**Authors:** Emily A. Kaip, Nicole Y. Nguyen, Jennifer M. Cocohoba

**Affiliations:** 1 Department of Pharmaceutical Services, University of California, San Francisco Medical Center, 505 Parnassus Avenue, San Francisco, CA 94143 USA; 2 Department of Clinical Pharmacy, University of California, San Francisco School of Pharmacy, 521 Parnassus Ave, CA 94117 San Francisco, Box 0622, USA

**Keywords:** HIV, AIDS, Bariatric surgery, Antiretroviral, Roux-en-y gastric bypass, Sleeve gastrectomy

## Abstract

**Purpose:**

Human immunodeficiency virus (HIV)–related mortality has decreased secondary to advances in antiretroviral therapy (ART), and the incidence of obesity in this population is increasing. Bariatric surgery is an effective method of weight loss, though changes in the gastrointestinal tract may affect ART absorption and virologic suppression. Existing data are limited to case reports studying outdated therapeutic regimens; studies evaluating modern ART regimens are needed. The objective of this study was to determine if undergoing bariatric surgery impacts HIV virologic failure rate at 12 months post-surgery and to characterize the failure population.

**Materials and Methods:**

This retrospective case series included adults with virologically suppressed HIV on ART who underwent roux-en-y gastric bypass (RYGB) or sleeve gastrectomy (SG) surgery between 2000 and 2019 (*n*=20) at one of three medical centers within one academic medical system. The primary outcome was proportion of patients with ART failure at 12 months post-surgery. Select additional data collected included CD4+ count, metabolic parameters, postoperative complications, and medication non-adherence.

**Results:**

A total of 18 patients were included in this analysis. Seventeen of 18 patients (94%) maintained virologic suppression within 12 months post-surgery. There were no significant changes in CD4+ counts before and after surgery. The one failure was an African American woman who underwent sleeve gastrectomy surgery. This patient’s baseline viral load was undetectable and CD4+ count was 263 cells/mm^3^.

**Conclusion:**

Undergoing bariatric surgery did not increase virologic failure rate in a small cohort of persons living with HIV, and ART non-adherence was associated with virologic failure.

**Graphical abstract:**

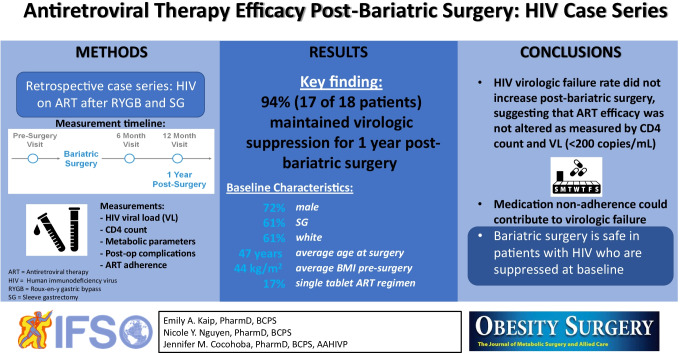

## Introduction

Advances in antiretroviral therapy (ART) have significantly prolonged acquired immunodeficiency syndrome-free survival rates in persons living with human immunodeficiency virus (HIV). With longer life expectancies, these persons become more prone to comorbidities associated with the aging population, such as obesity. A 2015 study estimated that the prevalence of obesity in men living with HIV engaged in medical care is about 19%, that of women is 42% [1], and most patients will gain weight during HIV infection and while on antiretroviral therapy [2–4]. Obesity predisposes patients to metabolic and cardiovascular disease, which are leading causes of death in patients living with HIV [5].

Bariatric surgery is an effective method of managing obesity and has been associated with improvements in obesity-related comorbidities such as diabetes mellitus [6] and obstructive sleep apnea [7]. Two common weight-loss surgeries are roux-en-y gastric bypass (RYGB) and sleeve gastrectomy (SG), both of which can be performed laparoscopically. During these procedures, the surface area of the stomach (and intestine in RYGB) is physically altered and the pH of the stomach increases as its size shrinks, becoming closer to that of the small intestine [8].

Changes in the pH and surface area of the gastrointestinal (GI) tract after surgery may affect HIV virologic suppression via altered ART absorption. Some antiretrovirals are absorbed in the duodenum, a piece of the intestine that is “bypassed” during RYGB surgery [8]. Additionally, select antiretrovirals depend on an acidic environment for optimal absorption [8], which may be impacted by surgery itself as well as acid suppression prescribed for ulcer prevention post-operatively.

Existing data on the efficacy of ART in persons living with HIV who have undergone bariatric surgery are limited to small-scale case reports that study outdated therapeutic regimens. Based on these case reports, virologic suppression was maintained after bariatric surgery in most subjects [9–14], though one prospective study of HIV patients undergoing SG found higher rates of virologic failure [15]. Of note, these studies included small sample sizes ranging from 1 to 17 subjects and studied antiretrovirals that are no longer used first line.

The primary objective of this study was to determine if undergoing RYGB or SG surgery affects efficacy of more modern ART regimens as measured by HIV virologic failure rate within 12 months following surgery, and to characterize the patients who suffered virologic failure.

## Materials and Methods

### Patients

This retrospective case series included adults with a diagnosis of HIV who were virologically suppressed on ART and underwent RYGB or SG between 2000 and 2019. Patients were engaged in care at either a bariatric surgery clinic or HIV clinic at one of three medical centers within the same academic health system. Patients were evaluated for RYGB or SG candidacy by standard inclusion criteria set forth by the American Society for Metabolic and Bariatric Surgery (ASMBS). Children under 18 years of age, pregnant women, and prisoners were excluded, as well as those with documented antiretroviral non-adherence in order to capture the effect of surgery on virologic failure.

### Data Collection

This study was approved by the local Institutional Review Board (IRB) and was enrolled in an IRB registry for multi-site studies within its academic health system. All patients fulfilling the pre-specified inclusion criteria were evaluated via retrospective electronic chart review. All parameters were assessed at three time points: the final office visit prior to surgery, and at 6- and 12-month follow-up visits post-surgery (plus-or-minus a 3-month range). Baseline demographic characteristics including age, race, and sex were collected for all patients. Other data collected from pre-surgery and post-surgery visits included the following: HIV viral load, CD4+ count, body weight, height, and metabolic parameters including hemoglobin A1c (A1c), serum glucose, and lipid panel values (low-density lipoprotein (LDL)-cholesterol, high-density lipoprotein (HDL)-cholesterol, triglycerides, and total cholesterol). Postoperative complications, acid suppression therapy usage (including antacids, histamine-2 receptor antagonists (H2RAs), and proton pump inhibitors (PPIs)), reasons for ART regimen changes either before or after surgery, medication non-adherence, and antiretroviral resistance were also recorded. The incidence of major and minor post-operative complications was identified per ASMBS Standardized Outcomes Reporting criteria [16]. All data were collected and managed using REDCap, a web-based application designed to support secure data capture.

### Primary and Secondary Outcomes

The primary outcome was ART failure within 12 months of surgery, defined by the US Department of Health and Human Services (DHHS) as failure to suppress or sustain an HIV viral load to less than 200 copies/mL [17]. Due to the long length of the study period, various assays were used to detect viral load including the Roche COBAS Ampliprep/COBAS TaqMan HIV-1 test with a lower limit of detection of 20 copies/mL most recently, branched DNA tests with a lower limit of detection of 75 copies/mL, and others. Secondary outcomes included characterizing those who suffered virologic failure compared to those with sustained virologic suppression in terms of metabolic parameters, CD4+ count, acid suppression use, postoperative complications, ART regimen changes, adherence, and resistance.

### Statistical Analysis

The primary outcome was quantified using descriptive statistics. Upon preliminary screening, the overall sample size was anticipated to be small and the study was unlikely to have sufficient power to detect a specific difference between types of surgery. Continuous variables were compared before and after surgery using paired *t*-tests, and between types of surgery using unpaired *t*-tests. Statistical significance was declared for two-sided *p*-values less than 0.05. Due to the low number of ART failures, descriptive statistics were used as appropriate to evaluate other secondary outcomes. All data analysis was conducted using Stata IC Version 13 (StataCorp, College Station, TX).

## Results

Twenty-seven persons living with HIV who had undergone RYGB or SG during the study period were identified among the three study sites. Eighteen patients were included in the primary outcome analysis and other analyses after nine patients were excluded for various reasons. Specifically, two patients did not have a pre-surgery HIV viral load available in the electronic health record (EHR), one patient did not meet the DHHS definition for virologic suppression pre-surgery (viral load >200 copies/mL), four patients did not have post-surgery viral load data available, and two patients had documented medication non-adherence.

### Baseline Characteristics

Overall, 7 patients (39%) underwent RYGB and 11 (61%) underwent SG. Patients were predominantly white (61%), male (72%), and the average (standard deviation, SD) age at surgery was 47.4 years (9.6 years). The mean (SD) BMI pre-surgery was 44.0 kg/m^2^ (5.0 kg/m^2^).

### Virologic Failure Rates and Descriptive Analysis of Failure Population

Of the 18 patients who met inclusion criteria and had pre-surgery and post-surgery viral load values available, 17 patients (94%) maintained virologic suppression for 12 months post-surgery. Baseline characteristics describing these patients can be found in Table 1. There were no significant changes in baseline CD4+ count following surgery and throughout the follow-up period. The average percentage of total body weight lost (%TWL) at 12 months post-surgery was 22% and the mean change in BMI was 10 kg/m^2^. Seventy-six percent of patients who underwent surgery were on acid suppression within 12 months post-surgery.

**Table 1:** Baseline characteristics stratified by type of surgery

	Total (*n*=18)	RYGB (*n*=7)	(SG (*n*=11)	*p*-value
Age at surgery, mean (SD)	47.4 (9.6)	45.3 (8.8)	48.8 (10.2)	0.46
Sex at birth, *n* (%)				0.95
Female	5 (28%)	2 (29%)	3 (27%)	
Race, *n* (%)				0.47
African American	7 (39%)	2 (29%)	5 (45%)	
White	11 (61%)	5 (71%)	6 (55%)	
Baseline BMI (kg/m^2^), mean (SD)	44.0 (5.0)	46.9 (6.1)	42.2 (3.2)	0.045
Baseline ART regimen, *n* (%)				0.83
Single tablet	3 (17%)	1 (14%)	2 (18%)	
Multiple tablet	15 (83%)	6 (86%)	9 (82%)	

*ART* antiretroviral therapy, *SD* standard deviation, *BMI* body mass index

Six patients (33%) encountered a total of nine postoperative complications within 12 months of surgery, one of whom suffered virologic failure (Table 3 in the Appendix). This patient suffered a late minor complication (vitamin or mineral deficiency) within 6 months of surgery.

Five patients (28%) had documented antiretroviral resistance prior to surgery, though none of these patients suffered virologic failure post-surgery. Reasons for preemptive ART regimen changes after surgery in the non-failure group included drug-drug interactions with acid suppression, inability to swallow certain tablets, or modernization to a newer ART regimen. A detailed record of baseline ART regimens and subsequent changes can be found in Table 4 in the Appendix.

The single patient who suffered virologic failure was a 43-year-old African American female who underwent sleeve gastrectomy surgery in 2015. The patient’s baseline CD4+ count was numerically lower (263 cells/mm^3^) than that of the average of non-failures (753 cells/mm^3^). Metabolic parameters (BMI, A1c, glucose, LDL, total cholesterol, HDL, triglycerides) were similar between types of surgeries at each time point (Table 2), and no meaningful differences were noted between the one patient who suffered virologic failure and the rest of the cohort. The %TWL was lower for the one failure patient compared to the rest of the non-failure population (18% vs. 23%). This patient was on a PPI post-surgery at the 6-month post-surgery visit, but not at the 12-month visit.

**Table 2:** Average change from baseline at 6 months and 12 months post-surgery

	RYGB (*n*=7)	SG (*n*=11)	*p*-value
CD4+ Count (cells/mm^3^), mean			
Pre-surgery	817.1	659.6	0.30
Post-surgery #1^a^	−72.9	+36.0	0.75
Post-surgery #2^b^	+259.4	−72.8	<0.001
Body mass index (kg/m^2^), mean			
Pre-surgery	46.9	42.2	0.045
Post-surgery #1	−11.8	−8.1	0.65
Post-surgery #2	−11.4	−9.1	0.44
Hemoglobin A1c (%), mean			
Pre-surgery	7.1	6.2	0.17
Post-surgery #1	−0.5	−0.3	0.43
Post-surgery #2	−1.2	−0.7	0.50
Plasma glucose (mg/dL), mean			
Pre-surgery	159.6	116.7	0.20
Post-surgery #1	−20.1	−17.3	0.15
Post-surgery #2	−30.8	−9.7	0.35
LDL (mg/dL), mean			
Pre-surgery	97.5	107.4	0.65
Post-surgery #1	+7.7	+0.6	0.88
Post-surgery #2	+4.7	−10.1	0.83
Total cholesterol (mg/dL), mean			
Pre-surgery	175.2	182.2	0.70
Post-surgery #1	+3.1	−4.3	0.98
Post-surgery #2	+4.0	−12.1	0.71
HDL (mg/dL), mean			
Pre-surgery	49	43	0.43
Post-surgery #1	−7.7	+3.7	0.37
Post-surgery #2	+0.2	+7.9	0.81
Triglycerides (mg/dL), mean			
Pre-surgery	142.3	159.3	0.62
Post-surgery #1	+17.7	−42.4	0.34
Post-surgery #2	−3.5	−50.6	0.36

*SD* standard deviation

^a^Post-surgery #1 = 6 months (± 3 months)

^b^Post-surgery #2 = 12 months (± 3 months)

For the single case with virologic failure, medical records noted a change from elvitegravir/cobicistat/emtricitabine/tenofovir disoproxil fumarate to elvitegravir/cobicistat/emtricitabine/tenofovir alafenamide. It is unknown what prompted this regimen change. Of note, the patient did not have documented adherence issues prior to surgery, and the patient did not develop any documented antiretroviral resistance in the 12 months following surgery. It also appeared this patient fell out of care around the time of when the study period concluded, as minimal lab values were available at this time point aside from HIV viral load and CD4+ T-cell count.

## Discussion

In this case series, undergoing bariatric surgery did not increase virologic failure rates in persons living with HIV who were virologically suppressed at baseline. These findings add to the existing body of evidence suggesting that persons living with suppressed HIV may successfully and safely undergo bariatric surgery without concern for virologic failure, the rate of which has been reported to be as high as 22.7% in the non-bariatric population [18].

It is challenging to draw conclusions about risk factors for HIV virologic failure after bariatric surgery from our case series. Acid suppression usage was not associated with virologic failure in the present study population, and the patient who experienced virologic failure was not on an ART regimen which would be affected by acid suppression. Avoidance of this risk factor may be embedded in practice, as most of the patients who were on an antiretroviral regimen where absorption would be impacted by acid suppression either had a preemptive regimen change or did not use acid suppression post-surgery. The patient who suffered virologic failure was not noted to develop antiretroviral resistance within 12 months following surgery. It is possible that resistance testing was never conducted, or that such results were not documented in the site’s EHR. Of note, there were instances where patients had no documentation of resistance, though the complexity of their baseline ART regimens may suggest otherwise. The single patient with virologic failure in our study cohort had a lower baseline CD4+ cell count; however, it is unclear whether this risk factor can be generalized to a broader surgery population. Other cross-sectional studies have found relationships between lower baseline CD4+ cell counts and virologic failure, but these are regimen and cohort specific [19].

In their single-center cohort study, Koethe and colleagues found that patients with HIV with a BMI in the range of 25 to 30 kg/m^2^ had a higher magnitude of immune reconstitution (measured by CD4+ count) compared to those outside this range 12 months after starting ART [20]. Because of these findings, it would not be surprising to see an increase in CD4+ counts after bariatric surgery. In the present study, CD4+ counts did not significantly change after surgery. That said, the average BMI of study subjects was 44 kg/m^2^ (5.0 kg/m^2^) at baseline, and 34 kg/m^2^ (5.6 kg/m^2^) after 12 months, which places most of these patients outside of the “optimal” BMI range established in the previous study.

Modern HIV antiretroviral regimens are potent, providing rapid and durable virologic suppression. While bariatric surgery has the potential to impact absorption and antiretroviral efficacy, results from our study cohort and other cohorts suggest that more traditional risk factors play a role in virologic failure. Our study excluded two patients who had documented non-adherence to their antiretroviral regimens. Both of these patients experienced treatment failure post-surgery. While our approach to our case series was to remove these patients to better illustrate relationships between surgery and virologic failure, they are an important reminder that medication adherence (and the potential resultant development of resistance) remains the primary driver of virologic failure.

This study is limited by its small sample size among three study sites, as well as the quality of chart notes and record keeping. The possibility for type II error exists, as the study was inadequately powered to detect a difference between groups in this niche population. Despite this, the present study serves as the largest published case series to date. Additionally, this study was not equipped to assess ART failure rates in the pre-integrase strand transfer inhibitor (INSTI) era. Raltegravir, the first available INSTI, was approved by the US Food and Drug Administration in 2007, but most of the present study data reflected surgeries completed in 2011 and later due to minimal electronic record availability. This study was also unable to assess lifestyle habits and adherence to post-surgery instructions, as well as long-term outcomes beyond 12 months following surgery.

The multi-site and retrospective nature of this study may have led to certain limitations, such as lack of consistency in timing of outcome measures and general differences in practice and patient populations. Though all sites used the same EHR (Epic, Epic Systems Corporation, Verona, WI), the timing of switching to electronic recordkeeping may have influenced the ease of data collection, as older data from the pre-EHR era was consistently more scarce.

Future studies to assess this niche population would ideally include a larger number of patients, which may be found through a national bariatric surgery registry. Additionally, emerging evidence suggests that ART, namely INSTI-based regimens and tenofovir alafenamide, has been associated with notable weight gain [21–25]. As such, future investigations may evaluate the frequency of bariatric cases secondary to weight gain caused by ART in persons living with HIV.

## Conclusion

In conclusion, bariatric surgery did not increase HIV virologic failure rate in this analysis. The present findings are consistent with existing literature with the additional advantage of including newer ART regimens. Bariatric surgery may be considered safe in persons living with HIV who are suppressed at baseline.

## Appendix

Tables

**Table 3:** Summary of post-operative complications suffered by time frame

	Early	Late
Minor	Nausea/vomiting requiring IV fluids (*n*=1) Surgical site SSTI (*n*=2)	Vitamin or mineral deficiency (*n*=3)
Major	Hospital readmission (*n*=1) ED visit/discharge (*n*=2)	--

Early—within 30 days post-surgery, late—greater than 30 days post-surgery

*IV* intravenous, *SSTI* skin and soft tissue infection, *ED* emergency department

3,

**Table 4:** Baseline ART regimens and regimen changes during study period

Surgery date	Pre-surgery	Post-surgery #1	Post-surgery #2
11/2011	ABC/3TC/AZT, RAL	-	-
11/15/11	EFV/FTC/TDF	-	-
1/16/13	ABC/3TC, ATV/r	-	-
2015	ABC/3TC, ATV	-	-
4/21/15	FTC/TDF, FPV, RAL	-	-
7/28/15	FTC/TDF, ATV/r	-	-
8/10/15*	EVG/c/FTC/TDF	-	-
9/8/15	FTC/TDF, ETR, RAL	-	-
6/17/16	ATV, ABC, DTG, 3TC	ABC, DTG, 3TC	-
9/13/16	FTC/TDF, DTG	-	-
1/12/17	ABC/3TC, DRV/c	-	-
5/15/17	FTC/TAF, DTG	-	-
8/22/17	FTC/TAF, DTG	-	-
8/25/17	FTC/TAF, DTG	-	-
11/17/17	FTC/TAF, DRV/r	FTC/TDF, DRV/r	FTC/TAF, DTG
4/16/18	ABC/DTG/3TC	(1) FTC/TAF, DTG (2) BIC/FTC/TAF	-
6/18/18	FTC/TAF, DRV/c	BIC/FTC/TAF	-
7/25/18	FTC/TAF, DTG	-	-

^*^Patient who suffered virologic failure

Regimens only listed in post-surgery 1 and 2 columns if regimen was changed. Separate dosage forms aside from ritonavir as a pharmacokinetic booster separated by comma. Post-surgery #1 = 6 months (± 3 months), post-surgery #2 = 12 months (± 3 months).

Abbreviations: *ABC* abacavir, *3TC* lamivudine, *ATV* atazanavir, *EFV* efavirenz, *FTC* emtricitabine, *TDF* tenofovir disoproxil fumarate, *RAL* raltegravir, *AZT* zidovudine, *r* ritonavir, *FPV* fosamprenavir, *EVG* elvitegravir, *c* cobicistat, *ETR* etravirine, *DTG* dolutegravir, *TAF* tenofovir alafenamide, *DRV* darunavir, *BIC* bictegravir

4
